# Abnormal visuo-vestibular interactions in vestibular migraine: a cross sectional study

**DOI:** 10.1093/brain/awy355

**Published:** 2019-02-12

**Authors:** Nadja F Bednarczuk, Angela Bonsu, Marta Casanovas Ortega, Anne-Sophie Fluri, John Chan, Heiko Rust, Fabiano de Melo, Mishaal Sharif, Barry M Seemungal, John F Golding, Diego Kaski, Adolfo M Bronstein, Qadeer Arshad

**Affiliations:** 1 Academic Department of Neuro-Otology, Division of Brain Sciences, Charing Cross Hospital Campus, Imperial College London, Fulham Palace Road, London, UK; 2 Department of Neurology, University Hospital Basel, Petersgraben 4, Basel, Switzerland; 3 Department of Neurology, Hospital das Clinicas da Faculdade de Medicina de Rebeirao Preto-USP, Campus Universitario s/n Riberao Preto, Sao Paulo, Brazil; 4 Department of Psychology, School of Social Sciences, University of Westminster, 115 New Cavendish Street, London, UK; 5 Department of Neuro-otology, Royal National Throat Nose and Ear Hospital, University College London, London, UK

**Keywords:** vestibular migraine, vestibular thresholds, visual motion adaptation, visuo-vestibular interaction

## Abstract

Vestibular migraine is among the commonest causes of episodic vertigo. Chronically, patients with vestibular migraine develop abnormal responsiveness to both vestibular and visual stimuli characterized by heightened self-motion sensitivity and visually-induced dizziness. Yet, the neural mechanisms mediating such symptoms remain unknown. We postulate that such symptoms are attributable to impaired visuo-vestibular cortical interactions, which in turn disrupts normal vestibular function. To assess this, we investigated whether prolonged, full-field visual motion exposure, which has been previously shown to modulate visual cortical excitability in both healthy individuals and avestibular patients, could disrupt vestibular ocular reflex and vestibular-perceptual thresholds of self-motion during rotations. Our findings reveal that vestibular migraine patients exhibited abnormally elevated reflexive and perceptual vestibular thresholds at baseline. Following visual motion exposure, both reflex and perceptual thresholds were significantly further increased in vestibular migraine patients relative to healthy controls, migraineurs without vestibular symptoms and patients with episodic vertigo due to a peripheral inner-ear disorder. Our results provide support for the notion of altered visuo-vestibular cortical interactions in vestibular migraine, as evidenced by vestibular threshold elevation following visual motion exposure.

## Teaser

Vestibular migraine is amongst the commonest causes of vertigo, but the regions underlying the development of symptoms including enhanced self-motion sensitivity and visually induced dizziness remain unknown. Bednarczuk *et al.* provide evidence for an abnormal interaction between visual and vestibular cortical regions in vestibular migraine.

## Introduction

Vestibular migraine accounts for 7% of diagnoses in neuro-otology and 9% of diagnoses in headache clinics (Dieterich *et al.*, 2016), thereby making it one of the most prevalent causes of episodic vertigo (Neuhauser, 2007; Dieterich *et al.*, 2016). Acutely, abnormalities of self-motion perception such as perceived whole-body spinning or rocking are common in these patients. On clinical examination, a nystagmus may be present, suggesting a degree of brainstem involvement, usually resolving between attacks (Bahra *et al.*, 2001; von Brevern *et al.*, 2004; Polensek and Tusa, 2010). In the interictal period, patients report spatial disorientation in addition to heightened self and visual motion sensitivity (Agarwal *et al.*, 2012). Currently, the diagnosis of vestibular migraine relies solely on clinical impression (Lempert *et al.*, 2012) as objective tests of vestibular function are typically normal, and if they are not, they are difficult to interpret as highlighted by the conflicting results of previous studies (Hong *et al.*, 2008; Roceanu *et al.*, 2008; Baier *et al.*, 2009; Murofushi *et al.*, 2009; Boldingh *et al.*, 2011; Taylor *et al.*, 2012; Kandemir *et al.*, 2013).

Despite the pathophysiology of vestibular migraine remaining largely unknown, recent findings have provided some preliminary insights. These include reports of abnormal vestibular thresholds during tilt (Lewis *et al.*, 2011; Wang and Lewis, 2016) and abnormal cortical interactions between visual and vestibular networks. The evidence for this latter point is provided by a neuroimaging study, which revealed altered metabolic activity in visual and vestibular cortical areas in two patients imaged during an acute episode of vestibular migraine (Shin *et al.*, 2014).

Based on the reviewed evidence, we propose to investigate whether impaired visuo-vestibular cortical interactions disrupt normal vestibular function in vestibular migraine patients. To address this, we assessed vestibular function through vestibular-ocular reflex and vestibular-perceptual thresholds during rotations before and after prolonged full-field unidirectional visual motion exposure. Previous work has demonstrated that implementing this visual-motion paradigm can modulate visual cortical excitability in healthy individuals (Lubeck *et al.*, 2017) and avestibular patients (Ahmad *et al.*, 2017), and as such represents an established method of probing visuo-vestibular cortical interactions (Brandt *et al.*, 1998). Further, this paradigm has also been shown to alter sensory thresholds in migraineurs (Drummond, 2002; Goadsby *et al.*, 2017). Accordingly, we predict that following visual motion exposure, vestibular migraine patients will exhibit altered vestibular ocular and perceptual thresholds due to abnormal visuo-vestibular cortical interactions.

## Materials and methods

### Participants

Fifiteen patients with vestibular migraine (mean age 42.0, range 21–61, 11 female) [see Table 1 for full patient details; note sample size derived from Cousins *et al.* (2013)], 15 patients with non-vertiginous migraine (implemented as a control for migraine) (mean age = 38.7, range 23–62, eight female) and 15 patients with benign positional paroxysmal vertigo (BPPV) (implemented as a control for vertigo) (mean age 44.7 range 26–66, seven female), were recruited from General Neurology and Neuro-otology Clinics (Charing Cross Hospital and The National Hospital for Neurology and Neurosurgery).

**Table 1:** Summary of vestibular migraine patient demographics and clinical details

VM patient	Gender	Age	DHI (total)	Trait anxiety	State anxiety	Duration of illness, months	Attack rate, attacks/month	Current medication (daily dose)
1	M	44	62	48	12	6	4	Candesartan (16 mg)
2	F	22	46	52	19	6	2	Paracetamol (PRN)
3	M	39	18	47	21	12	3	Propranolol (20 mg)
4	F	46	42	45	13	24	6	Amitriptyline (80 mg)
5	F	56	56	29	12	18	30	Amitriptyline (10 mg)
6	F	61	78	40	11	48	30	Amitriptyline (10 mg), propranolol (20 mg)
7	F	33	26	25	7	84	1	Ibuprofen (PRN)
8	M	59	30	65	13	36	12	Candesartan (8 mg)
9	F	37	14	61	21	36	30	Ibuprofen (PRN)
10	F	31	24	39	6	72	2	Ibuprofen (PRN)
11	F	32	46	34	13	12	30	Amitriptyline (10 mg), paracetamol (PRN)
12	F	44	24	32	6	48	3	Candesartan (8 mg)
13	F	59	98	60	6	9	3	Topiramate (100 mg)
14	M	53	44	35	6	48	4	Ibuprofen (PRN)
15	F	26	70	66	12	24	4	Paracetamol (PRN)
Summary	11 female / 4 male	42.8 (21–61)	45.2 (0–98)	45.2 (25–66)	11.8 (6–21)	32.3 (6–84)	10.9 (1–30)	

Summary values are mean (range). DHI = Dizziness Handicap Inventory; PRN = *pro re nata* (as needed); VM = vestibular migraine.

All migraineurs had active (an attack within the past year) vestibular migraine (mean days since last dizzy episode: 7.07; range 1–32) or non-vertiginous migraine (mean days since last headache: 44.47; range 1–365) at the time of testing. Note that this does not refer to the slight interictal symptoms patients report between attacks. All experiments were performed in the inter-ictal period. Patients were diagnosed by Consultant neurologists and conformed to either the Vestibular Migraine Barany consensus diagnostic criteria (vestibular migraine patients) (Lempert *et al.*, 2012) or the international classification of headache disorders (non-vertiginous migraine patients) [ICHD-3 Beta - The International Classification of Headache Disorders 3rd edition (Beta version), 2016]. The mean duration of dizziness symptoms in the vestibular migraine group was 32.3 months (range 6–84 months).

To control for any non-specific effects associated with dizziness in the vestibular migraine patients, we recruited posterior canal BPPV patients. These patients were recruited directly from Neuro-Otology clinics. The experiments in the BPPV patients were carried out 1 h after the repositioning manoeuvre (treatment) was performed. BPPV patients had dizziness symptoms for a mean duration of 19.5 months (range 3–82 months).

All patients had normal hearing (audiogram) and vestibular function (either caloric or rotational chair testing) to ensure no peripheral vestibular hypofunction. Vestibular migraine (Table 1) and non-vertiginous migraine patients were on various prophylaxis medications. Fifteen age matched healthy-controls (mean age: 41.8, range 19–64, seven females) were also recruited. All participants were naive to the experimental protocol. Written informed consent was obtained as approved by the local ethics research committee.

### Assessment of vestibular thresholds

**Figure 1:**
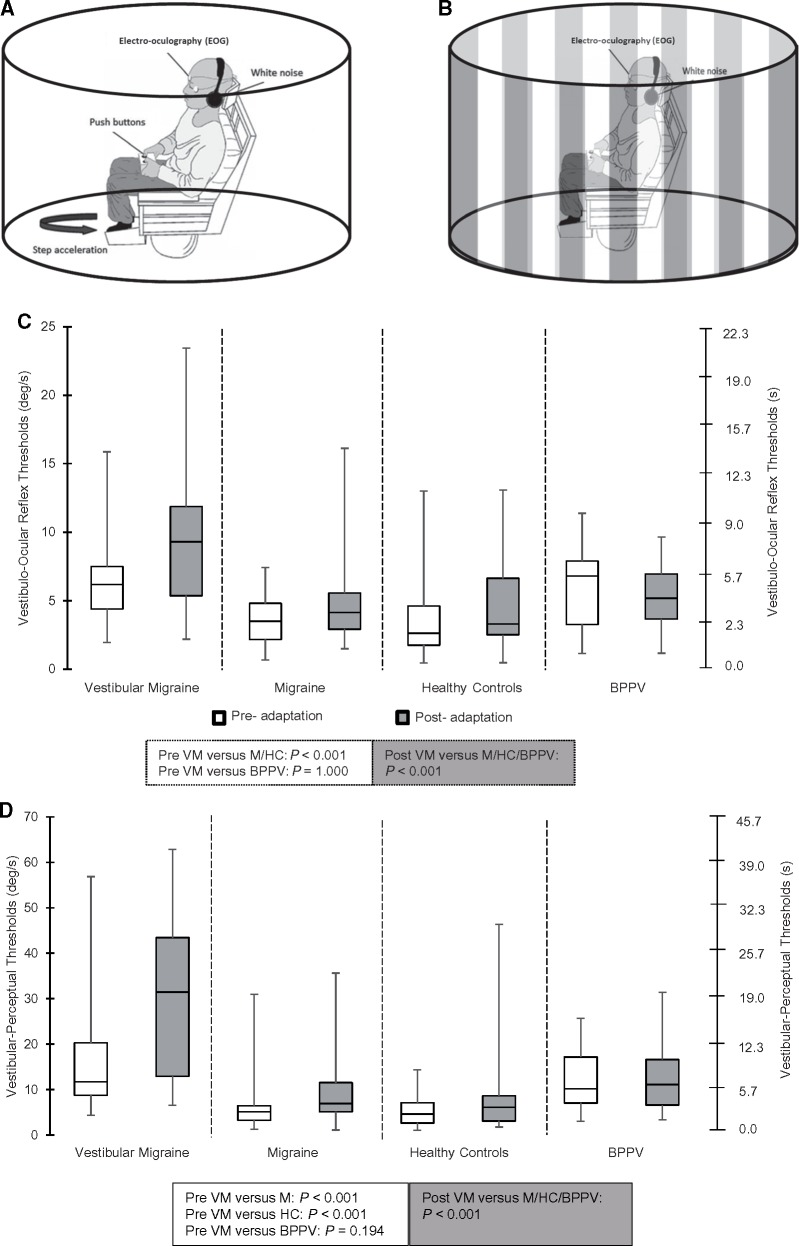
**Schematic representing the study methodology and main results.** (**A**) Vestibular-ocular reflex thresholds were measured at baseline with electro-oculography (EOG). Vestibular-perceptual thresholds were indicated with the help of button press in the subjects’ hands. White noise was amplified through speakers to mask sound cues. Six rotations were performed (three left, three right) in random order. These were repeated after visual motion exposure. (**B**) Visual motion (only rightward) was provided with a black and white striped curtain. The striped curtain encircled the subject and rotated for 5 min. Eye movements were monitored with electro-oculography to ensure subject viewing of the visual motion adaptation. Image adapted from Seemungal *et al.* (2008). (**C**) Summary of vestibular-ocular thresholds (VOR) in all experimental groups pre- (white boxes) and post-visual motion exposure (grey boxes). Vestibular-ocular reflex thresholds, as demonstrated by the mean nystagmus onset time (°/s) represented on the *y*-axis are shown for healthy controls, migraine, vestibular migraine and BPPV groups which are represented on the *x*-axis. The line in the middle of the box plot represents the median vestibular-ocular reflex threshold. The upper and lower boundaries represent the 25th and 75th percentile, respectively. The whiskers represent the 10th and 90th percentile. We observed a significant increase in vestibular-ocular reflex thresholds in the vestibular migraine group in comparison to healthy controls and migraineurs but not BPPV patients at baseline. Following visual motion exposure, we observed significantly elevated vestibular-ocular reflex thresholds in vestibular migraine when compared to healthy controls, non-vertiginous migraine and BPPV patients. No significant difference was observed between healthy controls and migraineurs either before or after visual motion exposure. (**D**) Vestibular-perceptual thresholds in all experimental groups pre- (white box) and post-visual motion exposure (grey box). Vestibulo-perceptual thresholds are shown on the *y*-axis by the mean perception onset time (or °/s) for healthy controls, migraine and vestibular migraine (VM) groups which are represented on the *x*-axis. Vestibulo-perceptual thresholds were significantly raised in the vestibular migraine group in comparison to healthy controls and migraineurs but not BPPV patients at baseline. Following visual motion exposure, thresholds in vestibular migraine patients were significantly higher compared to all three control groups. No difference was found between healthy controls and migraineurs either before or after visual motion exposure.

Participants were seated on a vibration-free motorized chair (Contraves) in total darkness with white noise delivered via a pair of chair-mounted radio-speakers to mask sound cues (Fig. 1A). Six yaw (horizontal plane) rotations (three rightward, three leftward; randomized order) were performed. Participants were provided with a rest period with lights turned on between rotations to reorientate. The chair accelerated at 0.3°/s^2^, increasing by 0.3°/s^2^ every 3 s (Cousins *et al.*, 2013; Kyriakareli *et al.*, 2013; Bednarczuk *et al.*, 2017). Participants indicated via a two-button press device (right and left) when they perceived the onset of direction-specific motion; trials with incorrect responses were repeated and excluded from threshold analysis. Vestibulo-perceptual thresholds were taken as the time elapsed between chair motion onset and button press, which could be converted to the chair velocity (°/s) at which point the button press occurred. Analysis was performed blind. For vestibular-ocular reflex thresholds, we recorded eye movements using electro-oculography. Time elapsing between onset of chair motion to the first nystagmic beat or consistent and sustained slow-phase eye movement deflection away from baseline (whichever was the earliest), provided the vestibulo-ocular reflex threshold. Calibration of the eye signal was performed using 20° saccadic targets to either the right or left of a central fixation target. Electro-oculography and chair tachometer velocity signals were sampled at 250 Hz. Analysis was performed using in-house software ‘Analysis’ (Kyriakareli *et al.*, 2013; Bednarczuk *et al.*, 2017) and blinded from the patient diagnosis.

### Visual motion

The chair was surrounded by a rotating 1.44-m diameter motorized optokinetic drum marked with black and white vertical stripes (Fig. 1B), and viewed at a distance of 0.72 m. The drum rotated rightwards at constant velocity (40°/s) around an Earth-vertical axis for 5 min when visual motion exposure was required. Participant’s compliance was monitored by online electro-oculography viewing of the resultant optokinetic nystagmus (Arshad *et al.*, 2014).

### Clinical questionnaires

Participants completed the Dizziness Handicap Inventory (Jacobson and Newman, 1990), a 25-item questionnaire to assess: (i) physical (seven questions, 28 points); (ii) functional (nine questions, 36 points); and (iii) emotional (nine questions, 36 points) factors associated with dizziness/unsteadiness (Jacobson and Newman, 1990). The Spielberger Trait and State Anxiety Inventory were also completed by participants to assess anxiety (validated measures) levels on a day-to-day basis (trait) and in response to the experimental protocol (situational anxiety; state) (Spielberger *et al.*, 1980; Spielberger, 2010).

To allow more detailed assessment of each vestibular migraine participant’s emotional state, in particular elements of low mood, the Hospital Anxiety and Depression Scale was also completed (Zigmond and Snaith, 1983). This scale includes 14 statements to assess both the presence of depression and anxiety, with scores ≥11 in either the depression or anxiety category reflecting a likely abnormality. Scores ≤7 are deemed as normal variants in either category.

Vestibular migraine patients were also asked to complete the Motion Sickness Susceptibility Questionnaire, as past findings have demonstrated increased susceptibility to motion sickness in this patient population, which could impact upon their vestibular thresholds (Cuomo-Granston and Drummond, 2010; Murdin *et al.*, 2015). This questionnaire assesses the experience of motion sickness in various exposures to motion, such as aircraft or cars, both during childhood and since the onset of symptoms in adulthood. This provides a total score reflecting vestibular migraine patient’s baseline sensitivity to motion, with higher scores reflecting greater sensitivity to motion (Golding, 1998, 2006).

### Experimental protocol

Both vestibular-ocular reflex and vestibulo-perceptual thresholds were assessed before (baseline) and after (adapted) 5 min of rightward full-field visual motion exposure. Following motion exposure, participants rested for one minute in the light to avoid any influence of optokinetic after nystagmus. Questionnaires were completed at the beginning of the experimental session, except the State component of the Spielberger Trait and Sate Anxiety Inventory, which was performed at the end.

### Statistical analysis

Repeated measures (4 × 2 × 2) ANOVA was performed for vestibular-ocular reflex and vestibular-perceptual thresholds. The factors considered were experimental Group (four factors: BPPV, vestibular migraine, migraine, healthy controls), Threshold (two factors: vestibular-ocular reflex, vestibular-perceptual), and Time (two factors: before and after visual motion exposure). *Post hoc t*-tests were performed throughout using Bonferroni corrections.

### Data availability

Data are available from the corresponding authors on request.

## Results

### Overview

In all participants (healthy controls, migraine, vestibular migraine and BPPV), baseline vestibulo-ocular reflex and vestibulo-perceptual thresholds were symmetrical. Vestibular migraine and BPPV patients exhibited significantly elevated ocular and perceptual thresholds at baseline compared to both healthy controls and migraine. Following visual motion exposure, both ocular and perceptual thresholds significantly increased further in vestibular migraine patients only. Post visual motion adaptation thresholds remained unchanged in BPPV patients, non-vertiginous migraineurs and healthy controls.

Repeated measures ANOVA revealed a significant main effect for experimental Group [*F*(3,27) = 38.478, *P < *0.001], Threshold type [*F*(1,29) = 196.654, *P < *0.0001] and Time [*F*(1,29) = 43.394, *P < *0.001]. There were also a significant 2-way interaction between Group × Time [*F*(3,27) = 11.529, *P < *0.0001], Group × Threshold [*F*(3,27) = 12.41, *P* < 0.0001] and, Time × Threshold [*F*(1,29) = 15.228, *P < *0.001]. A 3-way interaction between Group × Threshold × Time revealed a trend towards significance [*F*(3,27) = 2.32, *P* = 0.09].

### Vestibular-ocular reflex thresholds

**Figure 2:**
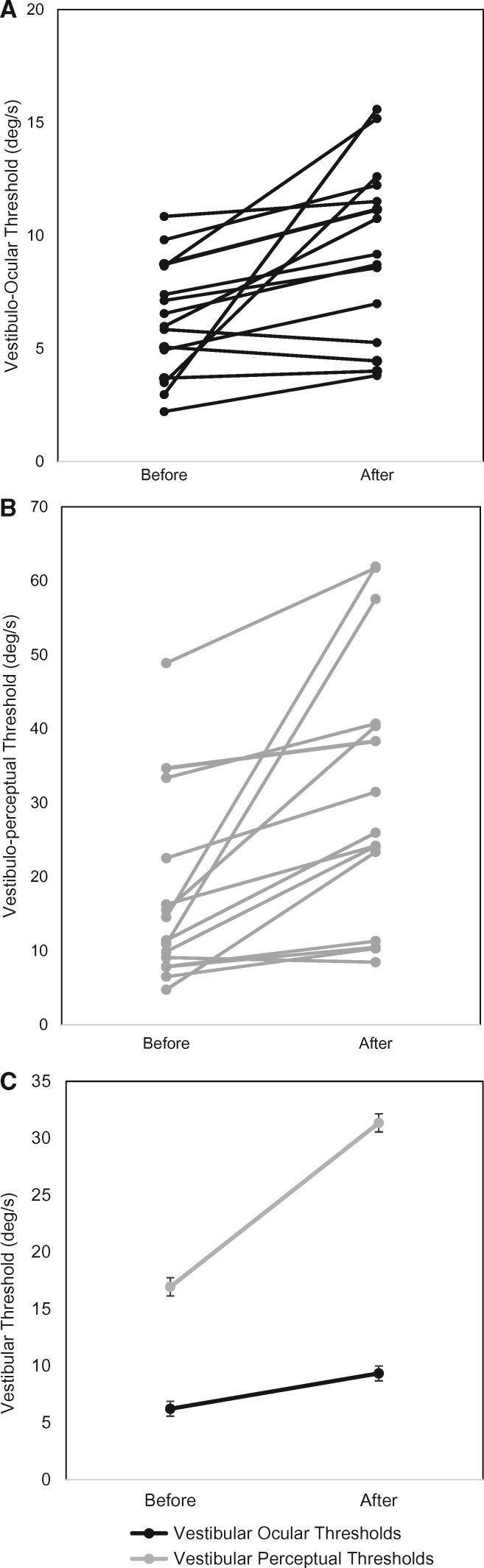
**Individual changes of thresholds in the vestibular migraine group before and after visual motion exposure.** (**A**) Individual changes in the vestibular migraine group for vestibular-ocular reflex thresholds. On the *y*-axis we represent the vestibular-ocular reflex threshold (°/s) and on the *x*-axis the time, either before or after visual motion exposure. (**B**) Individual changes in the vestibular migraine group for vestibulo-perceptual thresholds. (**C**) Group data for the vestibular migraine patients. On the *x*-axis we represent the condition, either the mean vestibular-ocular threshold (black line) or the vestibular-perceptual threshold (grey line) before and after visual motion exposure. On the *y*-axis we represent the vestibular threshold in degrees/second. As illustrated, both vestibular-ocular and vestibular perceptual thresholds became significantly raised following visual motion exposure. Error bars denote standard error.

Baseline vestibular-ocular reflex thresholds were significantly raised in vestibular migraine compared to healthy controls (*P < *0.01, *t*-test), and migraineurs (*P < *0.01, *t*-test) (Fig. 1C) but not BPPV patients (*P > *0.05). No difference was observed between controls and migraineurs (*P > *0.05). However, BPPV vestibular-ocular reflex thresholds were significantly higher at baseline when compared to controls and migraineurs (*P < *0.01, *t*-test). Following visual motion exposure, vestibular-ocular reflex thresholds in vestibular migraine patients became significantly further raised compared to baseline measures (*P < *0.001, *t*-test) and also when compared to healthy controls (*P < *0.001, *t*-test), non-vertiginous migraineurs (*P < *0.001; *t*-test) and BPPV patients (*P < *0.01) (Figs 1C and 2A). No differences were observed between controls and non-vertiginous migraineurs (*P > *0.05). There was no significant difference between the adapted vestibular-ocular reflex thresholds of BPPV patients when compared to both controls and non-vertiginous migraineurs (*P > *0.05, *t*-test). Critically, vestibular-ocular reflex thresholds in BPPV patients did not significantly change from baseline following the exposure to visual motion (*P > *0.05, paired *t*-test). There was no relationship between the time since the last episode of vestibular migraine and vestibular-ocular thresholds both before (R^2 ^= 0.373, *P > *0.05) and after (R^2 ^= 0.086, *P > *0.05) visual motion exposure.

### Vestibular-perceptual thresholds

Baseline perceptual thresholds were significantly raised in vestibular migraine compared to healthy controls (*P < *0.001) and migraineurs (*P < *0.01) (Fig. 1D) but not BPPV patients (*P > *0.05). No difference was observed between healthy controls, migraineurs and BPPV patients (*P > *0.05). Following visual motion exposure, adapted perceptual thresholds in vestibular migraine patients became significantly further raised compared to baseline measures (*P < *0.001, *t*-test) and also when compared to those observed in controls (*P < *0.001), non-vertiginous migraine (*P < *0.001) and BPPV patients (*P < *0.01) (Fig. 1D and 2B). No differences were observed between healthy-control and migraine patients (*P > *0.05). BPPV perceptual thresholds were significantly raised in comparison to controls (*P < *0.05 *t*-test), but not to non-vertiginous migraineurs (*P > *0.05, *t*-test). Similarly, to vestibular-ocular reflex thresholds, perceptual thresholds in BPPV patients remained unchanged following visual motion exposure (*P > *0.05, paired *t*-test). There was also no relationship between the vestibular perceptual thresholds, either before (R^2 ^= 0.0009, *P > *0.05) or after (R^2 ^= 0.076, *P > *0.05) visual motion exposure and the days since the last vestibular migraine episode.

### Error rate

**Figure 3:**
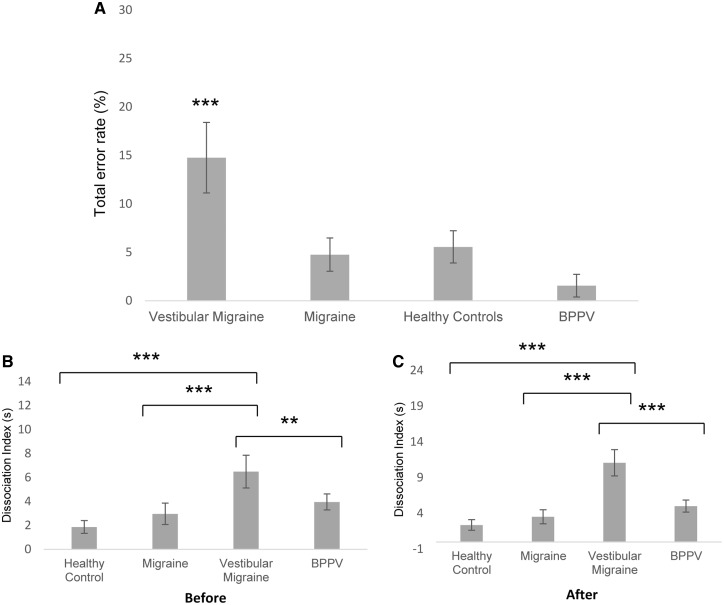
**Error rate in the task and vestibulo-ocular vestibulo perceptual dissociation in all groups.** (**A**) Total error rate comparison between vestibular migraine, migraine, healthy controls and BPPV patients. Total error rate encompasses all directional errors during the course of the experimental protocol and includes errors pre- and post-visual motion exposure. The total error rate was significantly higher in vestibular migraine patients in comparison to both non-vertiginous migraineurs, healthy controls, and BPPV patients. Error bars represent standard error of the mean (SEM). (**B**) The dissociation index (difference between vestibulo-perceptual and vestibulo-ocular threshold values) before visual motion exposure comparing controls, non-vertiginous migraineurs and BPPV patients to vestibular migraine patients. The dissociation of thresholds was significantly higher in the vestibular migraine group in comparison to the three controls group. (**C**) The dissociation index following visual motion adaptation comparing controls (^***^P < 0.001), non-vertiginous migraineurs (^***^P < 0.001) and BPPV patients to vestibular migraine patients (^**^P < 0.01). The dissociation of thresholds was significantly higher in the vestibular migraine group in comparison to the three controls group (^***^P < 0.001).

We computed the error rate in detecting direction-specific motion (i.e. pressing the right button when moving left). The error rate was solely elevated in vestibular migraine patients compared to non-vertiginous migraineurs, healthy controls and BPPV patients (*P < *0.001, *t*-test) (Fig. 3A).

### Inter-relationship between vestibular reflex and perceptual thresholds

Typically, perceptual thresholds are higher than ocular thresholds (Cousins *et al.*, 2013). Calculating the difference between vestibulo-perceptual and vestibular-ocular reflex thresholds allows you to gauge a dissociation index. At baseline, the dissociation index for vestibular migraine was significantly higher in comparison to controls and non-vertiginous migraineurs (*P < *0.05, *t*-test) (Fig. 3B). However, there was no significant difference between the baseline dissociation index for vestibular migraine patients and BPPV patients (*P > *0.05, *t*-test). Following visual motion exposure (Fig. 3C), the difference between perceptual and ocular thresholds further significantly increased only in the vestibular migraine group in comparison to all three control groups (*P < *0.01, *t*-test).

### The role of visual aura

Subgroup analyses of the vestibular migraine and non-vertiginous migraine group by means of the presence of visual aura (Ranson *et al.*, 1991) in both groups, revealed no significant differences in vestibular-ocular reflex or perceptual thresholds either before (*P > *0.05, *t*-test) or after (*P > *0.05, *t*-test) visual motion exposure.

### Clinical questionnaires

With respect to the clinical questionnaires (scores in Table 1 for vestibular migraine), we observed no relationship between either ocular or perceptual thresholds and anxiety levels or Dizziness Handicap Inventory, as assessed by the questionnaires in any of the experimental groups. The mean Dizziness Handicap Inventory score for controls and non-vertiginous migraine groups was 5.06 (range = 0–28), whereas the BPPV group had an average score of 40.9 (range 20–82) and 45.2 (range 14–98) for the vestibular migraine group. Further, trait anxiety scores averaged 45.2 in vestibular migraine (range 25–66), 44.4 (range 29–72) in non-vertiginous migraine patients, 32.3 (range 23–59) in healthy controls, and 39.4 (range 28–49) in BPPV patients. Mean state anxiety scores were 11.9 (range 6–21) in vestibular migraine patients, 9.7 (range 6–16) for non-vertiginous migraine patients, 7.7 (range 6–19) for healthy controls, and 10.9 (range 6–20) in BPPV patients. Additionally, the Hospital Anxiety and Depression Scale provided solely to the vestibular migraine patients highlighted a mean anxiety score of 8.87 (range 4–18) and a mean depression score of 5.01 (range 0–13). Importantly, no relationship was observed between the individual anxiety scores and patients ocular and perceptual thresholds, both before (ocular: R^2 ^= 0.0197, *P > *0.05; perceptual: R^2 ^= 0.0165, *P > *0.05) and after (ocular: R^2 ^= 0.072, *P > *0.05; perceptual: R^2 ^= 0.0013, *P > *0.05) visual motion exposure. Similarly, there was also no correlation between vestibular migraineurs depression scores and their ocular and perceptual thresholds either before (ocular: R^2 ^= 0.090, *P > *0.05; perceptual: R^2 ^= 0.018, *P > *0.05) or after (ocular: R^2 ^= 0.009, *P > *0.05; perceptual: R^2 ^= 0.063, *P > *0.05) visual motion exposure.

The mean motion sickness susceptibility in vestibular migraine patients was 13.91. No significant relationship between the motion sickness susceptibility and the vestibular-ocular thresholds was observed either before (R^2 ^= 0.186 *P = *0.11) or after (R^2 ^= 0.04, *P = *0.43) visual motion exposure. There was also no significant relationship with the susceptibility and vestibular perceptual thresholds prior to visual motion exposure (R^2 ^= 0.144, *P = *0.16), or following visual motion exposure (R^2 ^= 0.24 *P = *0.06) between the degree of motion sickness susceptibility and the vestibular perceptual threshold. Importantly, 5 of 15 vestibular migraine patients reported ‘feeling unwell’ and ‘symptomatic-dizzy’, at the end of the experiment protocol, a significant number given that none of the healthy controls, BPPV patients or non-vertiginous migraine patients reported any such symptoms (Fisher’s Exact Test two-tail *P = *0.001). Using independent sample t-tests, we observed no differences between either the vestibular ocular or perceptual thresholds in these five patients in the vestibular migraine group when compared to the other 10 patients either before or after visual motion exposure (*P > *0.05 independent sample t-test).

## Discussion

Vestibular migraine and BPPV patients exhibited abnormally elevated reflex and perceptual self-motion detection thresholds at baseline. Following visual motion exposure, reflex and perceptual thresholds were further raised only in the vestibular migraine group. Moreover, an exaggerated separation between ocular and perceptual thresholds (dissociation index) was observed at baseline in vestibular migraine and BPPV patients, which became more marked only in vestibular migraine patients after exposure to visual motion. Additionally, we identified a degree of spatial disorientation only in vestibular migraine patients characterised by more frequent errors in detecting self-motion direction (Fig. 3A).

### Baseline thresholds

Vestibular migraine patients exhibited elevated vestibular-ocular and perceptual thresholds during yaw (horizontal plane) rotations. This is in concordance with a previous finding that illustrated elevated thresholds for tilt perception during fixed-radius centrifugation in vestibular migraine patients (Wang and Lewis, 2016). Contrastingly, a separate study revealed that during dynamic roll tilt (frontal plane), vestibular migraine patients’ exhibit decreased perceptual thresholds (i.e. quicker to detect motion compared to controls) attributed to abnormal central canal-otolith integration (Lewis *et al.*, 2011). This discrepancy between our current observation and previous finding in the literature can be reconciled by considering the type of vestibular stimulus that patients were exposed to. Unlike previous studies, yaw rotations, as used in our current study, do not implicate canal-otolith interactions (Valko *et al.*, 2012). Critically, neither of the two prior studies introduced visual stimulation, an essential component of probing visuo-vestibular interactions and which we will turn to discuss in further detail below.

We propose that the observed changes in baseline thresholds may be attributable to either of the following two possibilities or a combination of both. First, vertiginous patients often report visually induced dizziness with everyday visual motion exposure, suggesting a disruption to normal visuo-vestibular cortical interactions in dizzy patients (Bense *et al.*, 2004; Agarwal *et al.*, 2012; Cousins *et al.*, 2014). Given that patients with vestibular dysfunction are hypersensitive to environmental visual stimuli (Bronstein, 1995; Guerraz *et al.*, 2001; Agarwal *et al.*, 2012), one possible explanation is that visual motion has a generalized effect and supresses vestibular thresholds. However, this is not supported by our findings, as in the BPPV group we only observed elevated thresholds at baseline but not on adapted thresholds post visual motion exposure (see below). An alternative explanation could be the presence of greater ‘noise’ in vestibular cortical networks in dizzy patients, equivalent to a ‘vestibular tinnitus’, impacting upon the task of detecting the direction of self-motion (Cousins *et al.*, 2013). Such a task can be conceptualized by applying the Drift Diffusion model (Mulder *et al.*, 2014), which predicts the time taken to reach a judgement (‘What direction am I moving in?’). This process allows for an interval for weighing up sensory information. When inappropriate ‘sensory noise’, in this case dizziness, is added to the decision-making process, it becomes more difficult to detect the motion signal, resulting in a longer time taken to reach a decision about the directionality of the movement (Mulder *et al.*, 2014). Accordingly, in vestibular migraine and BPPV, motion detection may be obscured by increased levels of ‘noise’ in visuo-vestibular networks, as further supported by data in vestibular patients (Jáuregui-Renaud *et al.*, 2008; Cousins *et al.*, 2013).

### Effect of visual motion exposure on vestibular thresholds

We demonstrate that visual motion exposure only modulated vestibular thresholds in vestibular migraine patients. Previous research has shown that prolonged unidirectional visual motion viewing modulates visual cortical excitability (Arshad *et al.*, 2014; Ahmad *et al.*, 2017; Lubeck *et al.*, 2017), as reflected by an increased probability of eliciting phosphenes in response to transcranial magnetic stimulation applied over both the early visual cortex (V1/V2) and middle temporal visual area (V5/MT) (i.e. hyper-excitability) (Arshad *et al.*, 2014; Ahmad *et al.*, 2017; Lubeck *et al.*, 2017). This altered excitability in the visual cortices following visual motion exposure may modulate vestibular cortical areas via visuo-vestibular cortical interactions (Brandt *et al.*, 1998), a notion explicitly supported by preliminary neuroimaging data in vestibular migraine. These data illustrate that during an acute vertiginous episode in vestibular migraine, there is reduced metabolism in the occipital cortex and increased metabolism in vestibular cortical areas (Shin *et al.*, 2014), highlighting the significance of visuo-vestibular cortical interactions in vestibular migraine (Agarwal *et al.*, 2012; Shin *et al.*, 2014).

Accordingly, implementing our visual motion exposure paradigm allowed us to functionally probe visuo-vestibular cortical interactions in vestibular migraine. Our data imply that adapted thresholds post visual motion exposure are not attributed to visual cortical hyperexcitability *per se* (i.e. no effect of visual motion adaptation upon vestibular thresholds in migraine, BPPV, or healthy controls with normal visuo-vestibular interactions) but rather, attributable to altered visuo-vestibular cortical interactions (i.e. visual motion adaptation increases vestibular thresholds only in patients with vestibular migraine). Functionally, these altered cortical interactions in vestibular migraine seemingly facilitate the hyper-excitable visual cortex to inhibit vestibulo-cortical areas, in turn attenuating self-motion perception and initiating top-down control of brainstem structures raising vestibular-ocular reflex thresholds (Battelli *et al.*, 2002; Arshad *et al.*, 2015; Ahmad *et al.*, 2017; Bednarczuk *et al.*, 2017).

More generically, the effects reported upon vestibular thresholds post visual motion exposure in the vestibular migraine patients argue against a cortical hyper-excitability theory (van der Kamp *et al.*, 1996; Aurora *et al.*, 1998; Chen *et al.*, 2011) and the notion of a habituation deficit (Coppola *et al.*, 2013; Ambrosini *et al.*, 2017). However, the observations of increased ocular and perceptual thresholds post visual motion exposure are compatible with the previously proposed central sensitization theory. This theory stipulates that migraineurs exhibit an enhanced responsiveness to sensory stimuli which can be driven by a diverse range of conditioning stimuli (Burstein, 2001; Dodick and Silberstein, 2006), namely visual motion in vestibular migraine patients. Accordingly, the net result of such hypersensitivity is the central amplification of noise (‘vestibular tinnitus’), which can then in turn explain the somewhat counter-intuitive observation of raised vestibular thresholds in our patient cohort following visual-motion exposure.

### Confounding influence of prophylactic medication on thresholds

Vestibular migraine and migraine patients were on various prophylactic medication at the time of testing (Table 1). Thus, it may be the case that these drugs potentially interfered with neurotransmitters and thereby modulated vestibular thresholds. However, we can rule out any potential modulatory effects of the drugs, given that there was no difference in baseline vestibular thresholds when comparing vestibular migraine and BPPV patients (none of whom were on any medication). Additionally, a difference in baseline thresholds was present when comparing vestibular migraine to non-vertiginous migraine (note that both patient groups were on medication).

## Conclusion

To summarize, we show abnormally elevated brainstem-mediated vestibular-ocular and cortically-mediated vestibular-perceptual thresholds at baseline and, critically, following visual motion adaption in vestibular migraine patients. We attribute these findings to altered visuo-vestibular cortical interactions in this condition.

## Abbreviation

BPPV: benign paroxysmal positional vertigo
